# Genetic Variation in Cell Death Genes and Risk of Non-Hodgkin Lymphoma

**DOI:** 10.1371/journal.pone.0031560

**Published:** 2012-02-07

**Authors:** Johanna M. Schuetz, Denise Daley, Jinko Graham, Brian R. Berry, Richard P. Gallagher, Joseph M. Connors, Randy D. Gascoyne, John J. Spinelli, Angela R. Brooks-Wilson

**Affiliations:** 1 Canada's Michael Smith Genome Sciences Centre, BC Cancer Agency, Vancouver, British Columbia, Canada; 2 Department of Medical Genetics, University of British Columbia, Vancouver, British Columbia, Canada; 3 Faculty of Medicine, University of British Columbia, Vancouver, British Columbia, Canada; 4 Department of Statistics and Actuarial Science, Simon Fraser University, Burnaby, British Columbia, Canada; 5 Department of Pathology, Royal Jubilee Hospital, Victoria, British Columbia, Canada; 6 Cancer Control Research, BC Cancer Agency, Vancouver, British Columbia, Canada; 7 Division of Medical Oncology and Centre for Lymphoid Cancer, BC Cancer Agency, Vancouver, British Columbia, Canada; 8 Department of Pathology and Centre for Lymphoid Cancer, BC Cancer Agency, Vancouver, British Columbia, Canada; 9 School of Population and Public Health, University of British Columbia, Vancouver, British Columbia, Canada; 10 Department of Biomedical Physiology and Kinesiology, Simon Fraser University, Burnaby, British Columbia, Canada; Instituto de Higiene e Medicina Tropical, Portugal

## Abstract

**Background:**

Non-Hodgkin lymphomas are a heterogeneous group of solid tumours that constitute the 5^th^ highest cause of cancer mortality in the United States and Canada. Poor control of cell death in lymphocytes can lead to autoimmune disease or cancer, making genes involved in programmed cell death of lymphocytes logical candidate genes for lymphoma susceptibility.

**Materials and Methods:**

We tested for genetic association with NHL and NHL subtypes, of SNPs in lymphocyte cell death genes using an established population-based study. 17 candidate genes were chosen based on biological function, with 123 SNPs tested. These included tagSNPs from HapMap and novel SNPs discovered by re-sequencing 47 cases in genes for which SNP representation was judged to be low. The main analysis, which estimated odds ratios by fitting data to an additive logistic regression model, used European ancestry samples that passed quality control measures (569 cases and 547 controls). A two-tiered approach for multiple testing correction was used: correction for number of tests within each gene by permutation-based methodology, followed by correction for the number of genes tested using the false discovery rate.

**Results:**

Variant rs928883, near miR-155, showed an association (OR per A-allele: 2.80 [95% CI: 1.63–4.82]; *p_F_* = 0.027) with marginal zone lymphoma that is significant after correction for multiple testing.

**Conclusions:**

This is the first reported association between a germline polymorphism at a miRNA locus and lymphoma.

## Introduction

Non-Hodgkin lymphoma (NHL) is a collection of different subtypes, each with different clinical presentation, preferred treatment regimens and prognosis. These subtypes appear to be derived from progenitor cells at different stages of B- and T-cell development. For example, mantle cell lymphomas are derived from pre-germinal centre B cells while follicular lymphomas arise from germinal centre B-cells [1]. The development of an active and specialized lymphocyte requires many steps [2], and each is controlled by apoptosis. If all requirements to proceed to the next level of development and activation are not met, lymphocytes enter a default program and are selected to die by apoptosis. This commonality interested us in testing the hypothesis that variation in cell death genes would affect risk of NHL.

Programmed cell death, the best characterized form of which is apoptosis, gives cells the ability to auto-destruct following specific triggers. Such triggers can be the presence or absence of a signal, depending on the cell context. Apoptosis has a central role in the development of the immune system, balancing the need for an effective immune response with the need to eliminate auto-reactive cells [2]. The crucial role of apoptosis in lymphoma was established by the discovery that the most common translocation in follicular lymphoma (FL), t(14;18), caused the inappropriate expression of *BCL2* by juxtaposing it to *IG* regulatory elements [3]. BCL2 is now known to be at the centre of a family of proteins with roles in apoptosis.

The number of BCL2-homology domains (or BH domains) determines whether the proteins in this family have anti-apoptotic or pro-apoptotic roles. Members with 4 BH-domains (BCL2, MCL1, and BCL-xL) have anti-apoptotic roles. Both the BH3-only members (including BIM, PUMA/BBC3, NOXA/PMAIP1, BID, BAD, BMF, BIK/BLK/NBK, HRK/DP5), which have only a BH3 domain, and the members with three BH domains (BAX and BAK) have pro-apoptotic functions [4]. Interactions between these diverse family members allow cells to respond to a variety of triggers, such as DNA damage, lack of B-cell receptor signaling from other cells, environmental toxins, or interleukin withdrawal. Tumour cells can acquire mutations that inactivate parts of this signaling network, causing tumourigenesis and drug resistance [5].

Seventeen candidate genes were selected following literature review and were chosen based on their role in apoptosis in lymphocyte development: *BAX*, *BCL2*, *BCL6* [6], *BCL10* [7], *BCL2L11*, *BBC3*, *BECN1* [8], *BMI1* [9], *FAS* [10], *MCL1*, *MDM2* [11], *PMAIP1*, *RFWD2* [12], *SKP2* [13], *miR-15a/16* [14], *miR-17-92* [15], and *miR-155* [16]. The coverage of known SNPs across each candidate gene at the time of initiation of the study was assessed by determining the ratio of the number of known SNPs to tagSNPs in each gene. 11 genes with low coverage were re-sequenced in DNA samples from 47 NHL cases. Targeted re-sequencing provided new, more thorough coverage of genetic information in candidate genes. SNPs were chosen for genotyping based on linkage disequilibrium relationships within the re-sequencing data and based on HapMap data. SNPs selected were genotyped in 797 cases and 790 controls and tested for association with risk of common NHL subtypes.

## Materials and Methods

### Study population

The study population has been previously described [17], [18], [19] and is summarized in **Table S1**. Briefly, all NHL cases diagnosed in British Columbia from March 2000 to February 2004, residing in the Greater Vancouver Regional District and Greater Victoria (Capital Regional District), and aged 20 to 79 were invited to participate. HIV-positive cases and cases with prior transplant were excluded. Categorization of ethnicity was based on self-reported ethnicity of each participant's four grandparents. Population controls were identified from the Client Registry of the British Columbia Ministry of Health and were frequency matched to cases by sex, age (within 5-year age groups), and area of residence in an approximate 1∶1 ratio. 828 cases and 848 controls completed at least part of a study questionnaire and 797 cases and 790 controls had DNA (**Table S1**). The study was approved by the joint University of British Columbia/British Columbia Cancer Agency Research Ethics Board. Written informed consent was obtained from all participants.

From the 1587 samples, DNA was derived from whole blood in 407 samples, lymphocytes isolated from blood in 782 samples, mouthwash in 24 samples and saliva in 48 samples. DNA extraction was done using the PureGene DNA isolation kit (Gentra Systems, Minneapolis, MN) following the manufacturer's instructions. DNA was quantified using PicoGreen (Molecular Probes, Invitrogen, Burlington, ON). Samples with limited amounts of DNA (326/1587 samples) were subjected to whole-genome amplification using the RepliG kit (QIAGEN, Mississauga, ON, Canada); these are hereafter referred to as “WGA” samples.

### Sequencing

Forty-seven NHL case samples (**Table S2**) were selected for SNP discovery via Sanger sequencing by a protocol previously described [20], based on age (preference was given to younger patients to enrich for genetics-based cases) and DNA quantity. Genomic sequences for use in primer design were obtained for each gene using Ensembl, with known SNPs annotated. Primers were designed using Primer 3 [21]. For all 17 candidate genes, 1.5 kb upstream of each gene's transcriptional start site was sequenced to identify genetic variation in promoter sequences near the gene; a subset of the candidate genes were subject to additional re-sequencing of all exons.

Genes were chosen for exon re-sequencing based on the depth of genetic variation already present in HapMap Phase II. For each gene, boundaries were selected by first capturing the gene with an extra 1.5 kb of sequence on either side of the gene within the browser window, and then extending the window boundaries until at least one more SNP in either direction was captured within the genomic region displayed. Genotypes were downloaded into Haploview [22]. Using Tagger [23] within Haploview [22], the set of tagSNPs required to capture the genotypes with *r* ^2^ = 0.8 was calculated. A gene was selected for exon re-sequencing if the ratio of #SNPs/#tagSNPs was less than 2. A ratio <2 indicates that there is less than 2-fold redundancy of tagSNPs in HapMap data, implying that re-sequencing may reveal as yet untagged variants in that gene. This cut-off was arbitrarily selected, as it can be interpreted to mean that, on average, a tagSNP represents at least one more SNP.

Uniform sequencing conditions were enabled by adding the -21M13F (TGTAAAACGACGGCCAGT) or M13R (CAGGAAACAGCTATGAC) extensions to the 5′ ends of the forward and reverse PCR primers, respectively. Primers and PCR conditions used are listed in **Table S3**. PCR reactions were followed by product cleanup using the Ampure bead system (Agencourt, Bioscience, Beverly, MA). A 2 uL aliquot of purified PCR product was cycle sequenced using Big Dye Terminator Mix V.3 at 1/24 chemistry in a total volume of 4 uL (Applied Biosystems, Foster City, CA) as described previously [20]. As a quality control measure, both forward and reverse directions were sequenced. Variants had to be present in both read directions to be considered valid. Sequences were assembled and inspected in Mutation Surveyor v3.24 (SoftGenetics, State College, PA). All new sequenced variants were deposited in dbSNP.

### TagSNP selection

Sequencing results were exported from Mutation Surveyor and imported into Haploview [22] for tagSNP selection using Tagger [23]. In addition, publicly available data from HapMap phase II was obtained as described above. To obtain tagSNPs representing both in-house re-sequencing data and public data, we used a strategy similar to one previously described [24]. Briefly, for each gene, tagSNPs were selected in four stages. We first selected tagSNPs for the SNPs that were present in both the sequencing results and the HapMap results, within the sequenced samples, using *r* ^2^ = 1 and a minor allele frequency cut-off (MAF) of 2% (set *a*). These tagSNPs were then preferentially selected in the next step, selecting tagSNPs with *r* ^2^ = 0.8 and MAF≥5% in HapMap samples from SNPs that were present in both the sequencing results and the HapMap results (set *b*). Third, tagSNPs were selected for all the SNPs found by sequencing, using the sequenced samples and force-including set *a*, with the same criteria as those used for set *a* (to make set *c*). Last, tagSNPs were selected from HapMap genotyping results, force-including set *b*, and using the same criteria as for set *b* (making up set *d*). The final genotyped set of SNPs was made up of the combination of sets *c*+*d*.

### Genotyping

The SNPs selected for this study were part of a larger Golden Gate assay (Illumina, San Diego, CA), which also contained SNPs from candidate genes in other pathways related to other hypotheses. SNPs predicted to fail assay design (most often due to being too close together) were replaced when possible by equivalent tagSNPs. In addition to tagSNPs, 51 ancestry-informative markers (AIMs) selected from Halder *et al.* [25] were included in the assay.

600 ng of each genomic or WGA DNA sample was arrayed into 18 96-well plates and sent to The Centre for Applied Genomics, the Hospital for Sick Children in Toronto, Canada. The resulting genotypes were assessed using Genome Studio version 2009.1 (Illumina, San Diego, CA). The first steps in genotype quality control (Q/C) were performed in Genome Studio; systems and databases in the laboratory of DD completed the final steps. Genotypes derived from WGA and genomic DNA were subjected to Q/C separately.

### Data quality control (SNPs)

For all genotypes, a GenCall Score cutoff of 0.25 was used, following Illumina recommendations for GoldenGate technology. The following SNPs were excluded: SNPs with GenTrain scores <0.4; SNPs with GenTrain scores between 0.4 and 0.7 and poor clustering (unexpected number of clusters, or clusters that were not well defined); SNPs with more than 3 clusters (which potentially indicate copy number variants); mono-allelic SNPs; SNPs with any genotype discrepancies between 53 pairs of duplicate samples; SNPs with call rate <95% in any sample type category (blood, mouthwash or saliva) were excluded for all samples in that category; one X-chromosome SNP that showed heterozygous calls in confirmed male samples; and SNPs out of Hardy-Weinberg equilibrium (*p*<0.001) in European-ancestry controls.

Of 178 tagSNPs selected for genotyping; 28/178 failed in the design phase, leaving 150 SNPs ordered for genotyping. Twenty-seven SNPs were excluded at the genotype quality control stage (12 SNPs were rejected by the genotyping centre upon initial inspection, 2 for low GenTrain scores, 5 for being potential copy number variants, 3 for being monoallelic, 3 for having a call rate <0.95, and 2 for having 1 error between duplicate genotypes). An additional 16 SNPs failed quality control only in WGA samples (7 for low GenTrain score and 9 for call rate <0.95), and 2 SNPs failed Q/C only in mouthwash or saliva samples. This left 123 SNPs (82%), listed in **Table S4**, for analysis in all non-WGA samples and 107 SNPs in both blood and WGA samples.

### Data quality control (samples)

WGA samples give different genotype intensities than non-WGA samples [26] and so were examined separately. SNPs were excluded from analysis in WGA samples only if they passed the above criteria but showed any discrepancies in genotype between 52 pairs of WGA sample and matched pre-WGA samples.

Samples with sex discrepancies (Qu *et al.*, *in press at Frontiers in Statistical Genetics and Methodology*) were excluded as they could have been subject to sample swaps during processing, as well as samples with call rate <0.98.

DNA samples from 1587 individuals were genotyped, 326/1587 being WGA samples. 58 non-WGA samples and 115 WGA samples were removed during quality control (23 non-WGA and 1 WGA sample due to gender discrepancies, and 35 non-WGA and 114 WGA samples due to call rate below 98%), leaving 1203 non-WGA samples+211 WGA samples, or 1414 samples in total.

45/51 AIMs passed quality control. Using genomic controls methodology, which has been shown to be appropriate when a modest number of candidate genes are assessed [27], [28], [29], we found no evidence for population stratification. The calculated inflation factor estimate was λ = 1.0.

Self reported ethnicities were confirmed using identity by state allele sharing and Multi-Dimensional Scaling (MDS) plots using HapMap data as the reference. These analyses identified two previously unidentified sets of sib-pairs, and three individuals who self-identified as Asian but clustered close to the HapMap CEU reference cluster. Sib-pair relationships were subsequently confirmed after review of study records; the youngest sibling of each pair (both were non-WGA samples) was excluded from analyses, as excess relatedness within a group of cases is not appropriate for a population-based association study. For the three subjects whose self-reported ethnicity was Asian, a review of study records indicated one subject self-reported being of Chinese ancestry, and two self-reported as being of Iranian descent. The subject of Chinese descent (non-WGA sample) was removed from the analyses due to concerns of a possible sample swap; and for the two subjects of Iranian descent the ethnicity was changed to “other”. This left 1200 non-WGA samples and 211 WGA samples, or 1411 samples in total (**Table 1**).

**Table 1:** Characteristics of samples used in genetic association analysis.

	Cases (%)	Controls (%)
**Gender**		
Male	416 (58%)	360 (52%)
Female	301 (42%)	334 (48%)
**Age group (years)**		
20–49	131 (18%)	172 (25%)
50–59	173 (24%)	153 (22%)
60–69	196 (27%)	185 (27%)
70+	217 (30%)	184 (27%)
**Ethnicity**		
Caucasian	569 (79%)	547 (79%)
Asian	66 (9%)	69 (10%)
South Asian	26 (4%)	31 (4%)
Mixed/Other	33 (5%)	29 (4%)
Unknown/Refused	23 (3%)	18 (2%)
**Pathology**		
**B-cell lymphomas**		
DLBCL	189 (26%)	-
FL	205 (29%)	-
MZL/MALT	78 (11%)	-
MCL	43 (6%)	-
SLL/CLL	39 (5%)	-
LPL	40 (6%)	-
MISC BCL	54 (8%)	-
**T-cell lymphomas**		
MF	38 (5%)	-
PTCL	24 (3%)	-
MISC TCL	7 (1%)	-
**Total**	717 (100%)	694 (100%)

DLBCL = Diffuse Large B-Cell Lymphoma, FL = Follicular Lymphoma, MZ/MALT = Marginal Zone lymphoma/Mucosa-Associated Lymphoma Tissue lymphoma, MCL = Mantle Cell lymphoma, SLL = Small Lymphocytic Lymphoma, LPL = Lymphoplasmacytic Lymphoma, Misc. B-cell = Miscellaneous B-cell lymphoma, MF = Mycosis Fungoides, PTCL = Peripheral T-Cell Lymphoma, Misc. T-cell = Miscellaneous T-cell lymphoma.

After all quality control, 1116/1411 samples (569 cases and 547 controls) of European ancestry were included in statistical analysis.

### Statistical analysis

Statistical analysis was performed using SVS Suite 7 (Golden Helix, Bozeman, MT). Logistic regressions under an additive model were fit for diffuse large B-cell lymphoma (DLBCL), follicular lymphoma (FL), marginal zone lymphoma (MZL), mantle cell lymphoma (MCL), all B-cell NHLs and all T-cell NHLs. In all subtype analyses, selected cases were compared to all controls. European-ancestry samples were used for analysis; other ethnicities (Asian, south-east Asian and “other”) were tested only for SNPs that showed association in European-ancestry samples. For each SNP, we calculated *p*-values for the model including the SNP of interest vs. the basic model (which accounted for 5-year age groups, sex, and region of residence). To test for genetic interaction between SNPs (in this example SNP A and SNP B), we tested a logistic regression model including an interaction term SNPA*SNPB against a model including the effects of SNP A, SNP B, 5-year age groups, sex and region of residence, limiting the analysis to European-ancestry samples.

To correct for multiple testing within each gene, full scan permutation carried out in SVS (10,000 permutations) [30], gave an adjusted *p*-value (denoted here by *p_p_*). For each permutation, the program randomly assigns case status to genotypes and calculates the number of times a more extreme *p*-value could be found by chance in permutations of all SNPs in the set. This method is similarly conservative to Bonferroni adjustment [30]. Like other lymphoma genetic epidemiology researchers [31], [32], we have used a two-tiered approach to multiple testing correction. The smallest adjusted *p*-value of SNPs within each gene was taken to represent the gene, and then the Benjamini-Hochberg procedure [33], implemented in R version 2.11.1, was applied to control the false-discovery rate (FDR) across the 17 genes and obtain a corrected *p*-value (*p_F_*). Adjusted *p*-values<0.05 were considered statistically significant. No multiple-testing correction was implemented for the few genetic interaction tests.

## Results

### Re-sequencing for SNP discovery

**Table 2** lists the candidate genes. Eleven of the 17 genes were selected for re-sequencing in 47 samples because they failed the criterion of #SNPs/#tagSNPs>2. Genes ranged from having a complete lack of genotyped SNPs in HapMap (for example the 10 kb gene *BMI1*) to densely genotyped genes such as *RFWD2* (which had 228 SNPs in HapMap and a #SNPs/#tagSNPs ratio of 7.9) (**Table 2**). Re-sequencing yielded 155 single nucleotide variants (**Table S5**), of which 40% was previously reported in dbSNP (build 127). Small insertions/deletions were as common as SNPs (40% vs. 39%, respectively). If one was to look only at relatively common SNPs (i.e. minor allele frequency (MAF) ≥5%), 72% of variants observed were previously reported at the time the sequencing results were analyzed, showing that most of the new information gathered by re-sequencing lay in rare variants. Eighty-eight tagSNPs were needed to capture the in-house sequencing data at *r* ^2^ = 1, with the exception of singletons which were not included in the next phase of this study. An *r* ^2^ of 1 was selected as the genetic information gained from sequencing was potentially more relevant because it was derived from lymphoma patient samples. Only 19/88 of these tagSNPs overlapped with tagSNPs used to capture the HapMap data, indicating that the information obtained is non-redundant with that in online databases and that the additional in-house sequencing produced novel genetic information valuable for representing (tagging) genetic variation in some of the candidate genes.

**Table 2:** Candidate genes.

Candidate Gene	#HapMap SNPs/#tagSNPs^1^	# novel SNPs found by re-sequencing^2^	# SNPs genotyped	# SNPs analyzed
***BAX***	1.40	4	8	7
***BCL2***	***2.76***	0	21	21
***BCL6***	1.54	12	12	11
***BCL10***	***2.25***	2	9	7
***BCL2L11***	***2.46***	1	11	7
***BBC3***	1.00	11	7	3
***BECN1***	1.00	3	2	1
***BMI1***	*	13	2	2
***FAS***	***3.14***	9	17	14
***MCL1***	1.00	7	11	7
***MDM2***	1.64	8	8	7
***PMAIP1***	1.00	9	9	6
***RFWD2***	***7.86***	6	10	10
***SKP2***	***2.90***	1	14	11
***MIR15A***	*	2	3	3
***MIR1792***	*	4	3	1
***MIR155***	*	0	3	3

*No SNPs were reported in HapMap.

1 Genes with ratio >2 are marked in bold and only had upstream regions re-sequenced.

2 Novel SNPs defined as not present in dbSNP build 127.

### Association results

For a summary of all analysis results see **Table 3**. One SNP showed an association that was strong enough to survive multiple testing correction both at the individual gene and multi-gene level. rs928883 (close to *miR-155*) was significantly associated with marginal zone lymphoma (OR: 2.80 [95% CI: 1.63–4.82]; *p_p_* = 0.0016; *p_F_* = 0.0272). rs2829803, located 4 kb away from rs928883 (*r* ^2^ = 0.41 between rs2829803 and rs928883), showed a weaker association with this same subtype (OR: 1.85 [95% CI: 1.12–3.08]). These associations were restricted to European ancestry samples, as there was no statistically-significant association in Asian samples or in South-Asian ancestry samples, although the power to detect such association was quite low in these smaller sample sets.

**Table 3:** Summary of results for the top-ranked SNP (ranked by *p_p_*) in each gene in European-ancestry samples for the major subtypes of non-Hodgkin lymphoma (SNP: odds ratio for additive model).

	DLBCL (n = 148)	FL (n = 165)	MZL/MALT (N = 55)	MCL (n = 40)	All B-cell (n = 523)	All T-cell (n = 45)
***BAX***	rs4645900: 0.62	rs4645900: 0.73	rs704243: 1.34	rs704243: 1.66	rs4645900: 0.66	rs11667229: 0.73
***BCL2***	rs4987873: 0.48	rs1026825: 1.35	rs1564483: 1.85	rs7226979: 1.37	rs4987852: 1.44	rs1801018: 0.57
***BCL6***	rs1474326: 1.30	BCL6_x10(3083)_A/T: 1.72	rs1474326: 0.86	rs2229362: 0.50	rs3733017: 1.34	rs1523474: 1.23
***BCL10***	rs12134420: 0.91	rs4949928: 1.25	rs4949927: 1.24	rs4949927: 1.63	rs4949928: 1.17	rs12134420: 1.19
***BCL2L11***	rs3761704: 1.25	rs10204044: 0.87	rs17041883:0.29*	rs17041868: 0.46	rs10204044: 0.89	rs17041868: 0.54
***BBC3***	rs45474992: 0.54	rs45474992: 0.68	rs884171: 0.90	rs45474992: 0.65	rs45474992: 0.66	rs884171: 0.73
***BECN1***	rs10512488: 1.36*	rs10512488: 0.93	rs10512488: 1.38	rs10512488: 1.52	rs10512488: 1.13	rs10512488: 1.16
***BMI1***	BMI1_UPSTR(-809)_C/T: 1.55	BMI1_UPSTR(-809)_C/T: 1.04	BMI1_UPSTR(-809)_C/T: 1.23	BMI1_UPSTR(-809)_C/T: 0.53	BMI1_UPSTR(-809)_C/T: 1.27	rs985000: 0.49
***FAS***	rs9658761: 1.34	rs2147420: 0.81	rs983751: 0.62	rs2147420: 0.69	rs978522: 1.19	rs9658761: 1.61
***MCL1***	rs6655975: 1.40	rs6655975: 0.77	rs35392872: 0.47	rs35392872: 0.59	rs35661734: 1.82	rs35661734: 2.63
***MDM2***	rs1695144: 0.45	rs769412: 0.68	rs3730536: 1.14	rs1695147: 1.41	rs769412: 0.85	rs937283: 1.45
***PMAIP1***	rs1041978: 1.22	rs1041978: 1.53	rs9957673: 0.67	rs1041978: 1.84	rs1041978: 1.35	rs1041978: 1.42
***RFWD2***	rs11587785: 1.27	rs12738115: 0.63	rs12738115: 0.63	rs6676805: 2.05*	RFWD2_UPSTR(-184)_C/G: 0.70	11587785: 2.37
***SKP2***	rs3804439: 0.82	rs2362973: 1.2	rs:17279275: 0.63	rs:17279275: 0.61	rs4440390: 1.16	rs10071838: 0.63
***MIR15A***	rs9535416: 0.81	rs9535416: 1.05	rs9535416: 0.71	rs2476391: 0.33	rs9535416: 0.91	rs2476391: 1.11
***MIR1792***	rs17642969: 0.89	rs17642969: 0.98	rs17642969: 0.74	rs17642969: 0.85	rs17642969: 1.03	rs17642969: 0.41*
***MIR155***	rs2829803: 0.87	rs928883: 1.35	rs928883: 2.80* ∧	rs2829803: 0.78	rs928883: 1.30 (	rs2829803: 1.61

All analyses are done against 547 European-ancestry controls.

*: *p_p_*<0.05,

∧ : *p_f_*<0.05. All p-values and number of samples in each category are listed in **Table S6**.

DLBCL: Diffuse Large B-Cell Lymphoma, FL: Follicular Lymphoma, MZ/MALT: Marginal Zone lymphoma/Mucosa-Associated Lymphoma Tissue lymphoma, MCL: Mantle Cell lymphoma.

Four other results indicated an association when the permuted *p*-values for correction within the gene were used (i.e. *p_p_*<0.05), but did not have *p_F_*<0.05. The SNP rs17041883 in *BCL2L11* for marginal zone lymphoma (OR: 0.29 [95% CI: 0.10–0.81]; *p_p_* = 0.033; *p_F_* = 0.280), rs6676805 in *RFWD2* for mantle cell lymphoma (OR: 2.05 [95% CI: 1.22–3.45]; *p_p_* = 0.048; *p_F_* = 0.823), rs17642969 close to *mir17-92* for T-cell lymphoma (OR: 0.409 [95% CI: 0.15–1.14]; *p_p_* = 0.047; *p_F_* = 0.800), and rs10512488 in *BECN1* for diffuse large B-cell lymphoma (OR: 1.36 [95% CI: 1.03–1.80]; *p_p_* = 0.034; *p_F_* = 0.578).

miR-155 has many known targets, including p53BP1 (the product of *TP53BP1*), AID (the product of *AICDA*) and AGTR1 [34]. We had genotypes for 4, 3 and 1 tagSNPs, respectively, for these genes, in this same sample set. These tagSNPs were selected using HapMap Phase II genotypes in the CEU population (*r* ^2^ = 0.8) for another study in our laboratory. We tested for interaction of each of these eight SNPs with rs928883. None of these eight SNPs were significantly associated with MZL. However, we found evidence for interactions of rs928883 near *miR-155* with rs714629 (*p* = 0.048) and rs9971686 (*p* = 0.051) in *AICDA* (interactions odds ratios are tabulated in **Table S7**). These two SNPs in *AICDA* are not in linkage disequilibrium with each other (*r* ^2^ = 0).

## Discussion

Association studies are often based on tagSNPs, which are usually selected from HapMap. TagSNPs derived from HapMap have been identified as non-transferable between populations in some cases [35] but transferable in others [36], particularly if tagSNPs are collected from different populations and merged to create a “cosmopolitan” set [36]. Our re-sequencing strategy, in which genes showing low redundancy of SNPs in HapMap were re-sequenced in individuals derived from the population of interest, ensures good representation of genetic variation in our genes of interest in these experiments. We note that between the time of sequencing and publication, continued activity in the sequencing of human genomes further populated databases; 33% of SNPs that were not reported in dbSNP build 127 are now in this public database (build 132).

rs928883, which is associated with MZL in our study, is located 2.3 kb upstream of *MIRN155*, in the second intron of *MIRN155HG*, the host gene of this 64 bp microRNA. It is in weak LD (*r* ^2^ of 0.37) with rs2829803, another SNP weakly associated with MZL. Replication of these findings is needed to validate the results, as our analysis used a relatively small group of 78 MZL samples.

The *Eμ-myc/miR-155* double hit mouse [37], which overexpresses both *myc* and miR-155, has an enlarged spleen, a decrease in white blood cells and high grade malignant lymphomas that are similar to human acute lymphoblastic leukemia or lymphoblastic lymphoma, beginning at 6 months of age. *miR-155* targets p53BP1 (p53-binding protein 1) [38], AID (activation-induced cytidines deaminase) [39], AGTR1 [34], and other genes. Known miR-155-targets are important players in lymphocyte development, marking miR-155 as an important connection point between these pathways.

miRNAs are ∼22 nucleotide RNAs that act as negative post-transcriptional regulators of gene expression by binding to imperfectly complementary sites on target mRNAs. 50% of miRNA genes are located in fragile sites or cancer-associated genomic regions [40], as well as being drastically reprogrammed in cancer and leukemia by copy number variation and differential expression [41]. Because miRNAs are short and highly conserved, mutations within miRNAs are expected to be uncommon. Consistent with this expectation, we found no polymorphisms in the transcribed region of the three miRNAs studied. Changes in allele frequencies of the SNPs located in miRNA target sites are potentially associated with human cancers [42]. In the past year, a few studies have explored the role of germline variants in microRNAs (particularly miR-146a, miR-196a and miR-499) in susceptibility to a variety of epithelial cancers, both familial [43] and sporadic – with both positive [44], [45] and negative reports [46], [47]. To our knowledge, however, no reports of tagSNPs or other SNPs near microRNAs affecting susceptibility to lymphoma or subtypes have been published thus far.

Although little is known about the role of miR-155 in MZL, its role has been investigated in other subtypes of NHL. High miR-155 expression is a marker of poor prognosis in HL and DLBCL [48], particularly in activated B-cell type DLBCL [49]. Its roles in modulation of cytokine production and AID regulation allow it to regulate the germinal centre response. Suppression of miR-155 in EBV-infected cells impedes entry into S phase and causes an increase in apoptosis [50]. The functions miR-155 has in lymphocytes and its documented effects in lymphoma certainly make it an interesting candidate gene for further study. As the causal variant may be another SNP in LD with rs928883, testing other variants in LD with this SNP could help to identify a variant that could affect miR-155's ability to repress its target mRNAs.

Morton *et al.* [31] examined risk of NHL with *BCL10*, *BCL2L11*, *BCL2*, *BAX* and *BCL6* and found associations of *BCL2L11* with FL, of *BCL6* with DLBCL and *BCL2* with MZL. Although we genotyped SNPs that either overlapped or were in high LD with these SNPs (r^2^>0.9), we did not replicate their findings. Two SNPs in *BCL2L11* previously reported to be associated with NHL by Kelly *et al.* [32], with one SNP associated with all NHL and the other also associated with CLL/SLL and FL in particular, were also genotyped and we were unable to replicate these results. rs4934436 in *FAS*, previously associated with NHL risk by Wang *et al.* [51], particularly with the DLBCL and FL subtypes, was not tagged at sufficiently high LD (*r* ^2^ = 0.6 with the haplotype CGG in the SNPs rs2234978/rs2147420/rs1571013, genotyped in this study) to test for replication. rs1056932 in *BCL6* was previously found to be associated with NHL, B-cell lymphomas and T-cell lymphomas [52]. This SNP was genotyped but did not show association with any NHL subtype tested. We examined SNPs in LD with rs2279744 (“SNP 309”) in *MDM2*, in female cases of DLBCL as suggested by Bond *et al.* [53] but found no association. Previous reports of no association with NHL risk with variants in *BCL2* [31], [54], [55], *BCL6* [56], [57], *BCL10* [31], [58], *BAX* [31], [54], *FAS* [59], and *MDM2* [60], [61], [62] are consistent with our negative findings. With the exception of the findings of Morton *et al.* [31] (which, based on our smaller sample size after quality control measures are applied, we had <80% power to replicate) and the association reported by Wang *et al.* [51] (for which the SNP was not adequately tagged in our study), our lack of replication of the other reported associations may be due to differences between populations, or could indicate false positive associations in those studies, as our study did have >80% power to replicate the reported associations.

Some studies have found that further classifying DLBCLs according to cell-of-origin subtypes Germinal-Centre B-cell like (GCB) and Activated B-Cell like (ABC) can clarify associations; for example, rs80031434 (G397C) was associated only with the GCB subtype of DLBCL [63]. We do not have comprehensive ABC and GCB classifications for our samples, however, and are unable to address this question. Another limitation of our study is the small number of cases for some NHL subtypes, particularly when analyzing non-European ethnicities. Finally, a large number of comparisons have been conducted and, while our multiple-testing correction methods are conservative, this nevertheless affects the power of this study.

In summary, we report an association for rs928883, located near miR-155, with marginal zone lymphoma. To our knowledge this is the first reported association between germline polymorphisms involving a miRNA gene and lymphoma, though the relevance of microRNAs to cancer in general is well established. We were not able to replicate some previously reported associations of SNPs in other apoptosis genes with subtypes of NHL. Replication of our finding in other studies is needed to confirm its significance.

## Supporting Information

Table S1 **Characteristics of the study population.** DLBCL = Diffuse Large B-Cell Lymphoma, FL = Follicular Lymphoma, MZ/MALT = Marginal Zone lymphoma/Mucosa-Associated Lymphoma Tissue lymphoma,MCL = Mantle Cell lymphoma, SLL = Small Lymphocytic Lymphoma, LPL = Lymphoplasmacytic Lymphoma, Misc. B-cell = Miscellaneous B-cell lymphoma, MF = Mycosis Fungoides, PTCL = Peripheral T-Cell Lymphoma, Misc. T-cell = Miscellaneous T-cell lymphoma.
(PDF)

Table S2 **Characteristics of the samples sequenced.** DLBCL = Diffuse Large B-Cell Lymphoma, FL = Follicular Lymphoma, MZ/MALT = Marginal Zone lymphoma/Mucosa-Associated Lymphoma Tissue lymphoma,MCL = Mantle Cell lymphoma, SLL = Small Lymphocytic Lymphoma, LPL = Lymphoplasmacytic Lymphoma, Misc. B-cell = Miscellaneous B-cell lymphoma, MF = Mycosis Fungoides, PTCL = Peripheral T-Cell Lymphoma, Misc. T-cell = Miscellaneous T-cell lymphoma.
(PDF)

Table S3 **PCR primers and reaction conditions.**
(PDF)

Table S4 **Variants available for testing after data cleaning.**
(PDF)

Table S5 **Variants discovered by re-sequencing.**
(XLS)

Table S6 Summary of results for top-ranked SNP (ranked by pp) in each gene in European-ancestry samples for the major subtypes of non-Hodgkin lymphoma (odds ratio for additive model, p-value* adjusted within gene and p-value* adjusted overall). All analyses are done against 547 European-ancestry controls. p-values<0.05 are in bold. DLBCL = Diffuse Large B-Cell Lymphoma, FL = Follicular Lymphoma, MZ/MALT = Marginal Zone lymphoma/Mucosa-Associated Lymphoma Tissue lymphoma,MCL = Mantle Cell lymphoma.
(XLS)

Table S7 **Interaction table displaying odds ratios for rs928883 and two AICDA SNPs, rs714629 and rs9971686, derived under an additive model.**
(PDF)
