# Supplementary material for: Genetic Variation in Cell Death Genes and Risk of Non-Hodgkin Lymphoma
Source: PLoS One. 2012 Feb 7;7(2):e31560. doi: 10.1371/journal.pone.0031560 (PMC3274532; doi:10.1371/journal.pone.0031560)
Supplement: Table S1 — Characteristics of the study population. DLBCL = Diffuse Large B-Cell Lymphoma, FL = Follicular Lymphoma, MZ/MALT = Marginal Zone lymphoma/Mucosa-Associated Lymphoma Tissue lymphoma,MCL = Mantle Cell lymphoma, SLL = Small Lymphocytic Lymphoma, LPL = Lymphoplasmacytic Lymphoma, Misc. B-cell = Miscellaneous B-cell lymphoma, MF = Mycosis Fungoides, PTCL = Peripheral T-Cell Lymphoma, Misc. T-cell = Miscellaneous T-cell lymphoma. (PDF) [file pone.0031560.s001.pdf]

Table S1 - Characteristics of the study population.

	Cases (%)	Controls (%)
**Gender**		
Male	463 (58%)	420 (53%)
Female	334 (42%)	370 (47%)
**Age group (years)**		
20-49	150 (19%)	206 (26%)
50-59	194 (24%)	169 (21%)
60-69	214 (27%)	206 (26%)
70+	239 (30%)	209 (26%)
**Ethnicity**		
Caucasian	625 (78%)	610 (77%)
Asian	80 (10%)	88 (11%)
South Asian	29 (4%)	37 (5%)
Mixed/Other	36 (5%)	36 (5%)
Unknown/Refused	27 (3%)	19 (2%)
**Pathology**		
**B-cell lymphomas**		
DLBCL	208 (26%)	-
FL	223 (28%)	-
MZL/ MALT	95 (12%)	-
MCL	47 (6%)	-
SLL/CLL	43 (5%)	-
LPL	43 (5%)	-
MISC BCL	62 (8%)	-
**T-cell lymphomas**		
MF	40 (5%)	-
PTCL	29 (4%)	-
MISC TCL	7 (1%)	-
**Total**	797 (100%)	790 (100%)

DLBCL = Diffuse Large B-Cell Lymphoma, FL = Follicular Lymphoma, MZ/MALT = Marginal Zone lymphoma/Mucosa-Associated Lymphoma Tissue lymphoma,MCL = Mantle Cell lymphoma, SLL = Small Lymphocytic Lymphoma, LPL=Lymphoplasmacytic Lymphoma, Misc. B-cell = Miscellaneous B-cell lymphoma, MF = Mycosis Fungoides, PTCL = Peripheral T-Cell Lymphoma, Misc. T-cell = Miscellaneous T-cell lymphoma.
