# Supplementary material for: Genetic Variation in Cell Death Genes and Risk of Non-Hodgkin Lymphoma
Source: PLoS One. 2012 Feb 7;7(2):e31560. doi: 10.1371/journal.pone.0031560 (PMC3274532; doi:10.1371/journal.pone.0031560)
Supplement: Table S2 — Characteristics of the samples sequenced. DLBCL = Diffuse Large B-Cell Lymphoma, FL = Follicular Lymphoma, MZ/MALT = Marginal Zone lymphoma/Mucosa-Associated Lymphoma Tissue lymphoma,MCL = Mantle Cell lymphoma, SLL = Small Lymphocytic Lymphoma, LPL = Lymphoplasmacytic Lymphoma, Misc. B-cell = Miscellaneous B-cell lymphoma, MF = Mycosis Fungoides, PTCL = Peripheral T-Cell Lymphoma, Misc. T-cell = Miscellaneous T-cell lymphoma. (PDF) [file pone.0031560.s002.pdf]

Table S2 - Characteristics of the samples sequenced.

	Cases (%)	Controls (%)
**Gender**		
Male	36 (77%)	-
Female	11 (23%)	-
**Age group (years)**		
20-49	23 (49%)	-
50-59	24 (51%)	-
60-69	-	-
70+	-	-
**Ethnicity**		
Caucasian	35 (74%)	-
Asian	10 (21%)	-
South Asian	2 (4%)	-
Mixed/Other	-	-
Unknown/Refused	-	-
**Pathology**		
**B-cell lymphomas**		
DLBCL	13 (28%)	-
FL	16 (34%)	-
MZL/ MALT	6 (13%)	-
MCL	2 (4%)	-
SLL/CLL	-	-
LPL	2 (4%)	-
MISC BCL	4 (9%)	-
**T-cell lymphomas**		
MF	1 (2%)	-
PTCL	-	-
MISC TCL	3 (6%)	-
**Total**	47 (100%)	

DLBCL = Diffuse Large B-Cell Lymphoma, FL = Follicular Lymphoma, MZ/MALT = Marginal Zone lymphoma/Mucosa-Associated Lymphoma Tissue lymphoma,MCL = Mantle Cell lymphoma, SLL = Small Lymphocytic Lymphoma, LPL=Lymphoplasmacytic Lymphoma, Misc. B-cell = Miscellaneous B-cell lymphoma, MF = Mycosis Fungoides, PTCL = Peripheral T-Cell Lymphoma, Misc. T-cell = Miscellaneous T-cell lymphoma
