# Supplementary material for: Genetic Variation in Cell Death Genes and Risk of Non-Hodgkin Lymphoma
Source: PLoS One. 2012 Feb 7;7(2):e31560. doi: 10.1371/journal.pone.0031560 (PMC3274532; doi:10.1371/journal.pone.0031560)
Supplement: Table S3 — PCR primers and reaction conditions. (PDF) [file pone.0031560.s003.pdf]

Table S3 - PCR primers and reaction conditions

Amplicon Name	Ann. Temp.	Enhancer*	Reaction times*	No. of cycles*
BAX_exon1	65			35×
BAX_exon2&3	65			35×
BAX_exon4	64			
BAX_exon5	61			
BAX_exon6	64			
BAX_exon7	65			35×
BAX_upstr_b	65			35×
BCL10_alt_upstr	62			35×
BCL10_upstr_b	58		10s ann	45×
BCL2_upstr_a	54	2×	5s ann, 10s ext	36×
BCL2_upstr_b	50	2×	5s ann, 10 s ext	36×
BCL6_exon1	63			
BCL6_exon10a	56			35×
BCL6_exon10b	54			35×
BCL6_exon2	63			
BCL6_exon3	65		10s ann	35×
BCL6_exon4	64			35×
BCL6_exon5a	62			
BCL6_exon5b	61			
BCL6_exon6	61			
BCL6_exon7	65			35×
BCL6_exon8	61		15s ann	
BCL6_exon9	65			35×
BCL6_upstr_a	59			35×
BCL6_upstr_b	59			35×
BECN1_exon1&2	61			
BECN1_exon10	59			35×
BECN1_exon11	59			
BECN1_exon12a	52			
BECN1_exon12b	52			
BECN1_exon3	61			
BECN1_exon5&6&7	64			35×
BECN1_exon8	62			
BECN1_exon9	61			35×
BECN1_upstr_a	56			35×
BECN1_upstr_b	54		15s ann	35×
BIM_upstr_a	57			35×
BIM_upstr_b	57			35×
BMI_exon10a	57			
BMI_exon10b	57			
BMI_exon10c	56			35×
BMI_exon10d	54			35×
BMI_exon1a	59		15s ann	
BMI_exon1b	50	2×	5s ann, 10 s ext	36×
BMI_exon2&3	56			
BMI_exon4&5	52			
BMI_exon6&7	59			
BMI_exon8&9	56			
BMI1_up_a	56			47×
BMI1_upstr_b	58	2×	5s ann, 10s ext	36×
FAS_alt_upstr_a	50			35×
FAS_alt_upstr_b	56			35×

FAS_upstr_a	60		10s ann	40×
FAS_upstr_b	50		10s ann	37×
MCL1_exon1a	63			
MCL1_exon1b	64			35×
MCL1_exon2	61			
MCL1_exon3b	65			35×
MCL1_upstr_a	64			35×
MCL1_upstr_b	63			35×
MDM2_alt_upstr	63			35×
MDM2_exon11a	61			35×
MDM2_exon11b	59			35×
MDM2_exon2	59			35×
MDM2_exon4	59			35×
MDM2_exon6&7	56			
MDM2_exon8	59			
MDM2_upstr_a	64			35×
MDM2_upstr_b	61			35×
miR15_upstr_A	50		10s ann	40×
miR15_upstr_B	51		10s ann	37×
miR155_loop	50	2×		36×
miR155_upstr	50	2×		
miR17_upstr_A	52			
miR17_upstr_B	51			36×
PMAIP1_exon1&2	65			35×
PMAIP1_exon3a	56			35×
PMAIP1_exon3b	54			35×
PMAIP1_exon3c	59			35×
PMAIP1_upstr_a	64			35×
PMAIP1_upstr_b	64			35×
PUMA_exon1	64			35×
PUMA_exon2	65			35×
PUMA_exon3	63			
PUMA_exon4a	64			35×
PUMA_exon4b	64			35×
PUMA_upstr_b	64			35×
RFWD2_alt_upstr_a	54			35×
RFWD2_alt_upstr_b	53			35×
RFWD2_upstr_a	56		10s ann	37×
RFWD2_upstr_b	61			35×
SKP2_alt_upstr	62			35×
SKP2_upstr_a	61			35×
SKP2_upstr_b	65			35×

*Unless conditions are specified in above table, all conditions are as described in Methods.

Amplicon Name	Forward primer
BAX_exon1	TGTAAAACGACGGCCAGTatgctcattggacagtcacg
BAX_exon2&3	TGTAAAACGACGGCCAGTctgctagggtcccagaagtc
BAX_exon4	TGTAAAACGACGGCCAGTtctttagtgtgcggtggatg
BAX_exon5	TGTAAAACGACGGCCAGTcttcgggtcttcatcctgag
BAX_exon6	TGTAAAACGACGGCCAGTaggggaggcaaagaattgac
BAX_exon7	TGTAAAACGACGGCCAGTaatgcccgttcatctcagtc
BAX_upstr_b	TGTAAAACGACGGCCAGTGCTTCAGCCCGGGAAT
BCL10_alt_upstr	TGTAAAACGACGGCCAGTGCTTCTTACACAGCGCCATA
BCL10_upstr_b	TGTAAAACGACGGCCAGTGCCTCATCCTACTTCCCTGTT
BCL2_upstr_a	TGTAAAACGACGGCCAGTTCCGCACTCCGTCGTC
BCL2_upstr_b	TGTAAAACGACGGCCAGTGCTGCCAGCGAAGGTG
BCL6_exon1	TGTAAAACGACGGCCAGTcgacgtcacagaggaggag
BCL6_exon10a	TGTAAAACGACGGCCAGTtgagcaatgacatctttcttgg
BCL6_exon10b	TGTAAAACGACGGCCAGTcaagacttcagtatgttgtcaaagaga
BCL6_exon2	TGTAAAACGACGGCCAGTggcaagctgcaactatcaaa
BCL6_exon3	TGTAAAACGACGGCCAGTgctaagccacctgctctgat
BCL6_exon4	TGTAAAACGACGGCCAGTaggggctgagtatcagtgct
BCL6_exon5a	TGTAAAACGACGGCCAGTaagtgtcactggaaggtcatct
BCL6_exon5b	TGTAAAACGACGGCCAGTgagggcctcaaacctgct
BCL6_exon6	TGTAAAACGACGGCCAGTgcctacacacagggaggaga
BCL6_exon7	TGTAAAACGACGGCCAGTcggccttagagccctacct
BCL6_exon8	TGTAAAACGACGGCCAGTGGAAAACACAAGTGGCCTCA
BCL6_exon9	TGTAAAACGACGGCCAGTCTGGCTGTGTGCTAATGAGG
BCL6_upstr_a	TGTAAAACGACGGCCAGTCCCCCAACTCAGCTTCAA
BCL6_upstr_b	TGTAAAACGACGGCCAGTCGCTTGAGGGATTAGATAGGG
BECN1_exon1&2	TGTAAAACGACGGCCAGTagcctccccattctctgc
BECN1_exon10	TGTAAAACGACGGCCAGTccttggccagaatgaagagt
BECN1_exon11	TGTAAAACGACGGCCAGTtgggaacaatctctggaagc
BECN1_exon12a	TGTAAAACGACGGCCAGTcctgccccgaacttacttaac
BECN1_exon12b	TGTAAAACGACGGCCAGTccagagctacaacatgccatc
BECN1_exon3	TGTAAAACGACGGCCAGTtcagatgccctcctgcttt
BECN1_exon5&6&7	TGTAAAACGACGGCCAGTagtaggtggaggaagcctct
BECN1_exon8	TGTAAAACGACGGCCAGTcgttcatagtgtggtgagagaga
BECN1_exon9	TGTAAAACGACGGCCAGTggtgagagtgggaaatgtcaa
BECN1_upstr_a	TGTAAAACGACGGCCAGTCAAGGGGAGATGGGAAATG
BECN1_upstr_b	TGTAAAACGACGGCCAGTCGGGATTTAACCATTTTG
BIM_upstr_a	TGTAAAACGACGGCCAGTAACAATCGCCTCGCCTTT
BIM_upstr_b	TGTAAAACGACGGCCAGTTCAGAGAAGTTCTGTCTGATTCG
BMI_exon10a	TGTAAAACGACGGCCAGTcctggagaagggtaagtagca
BMI_exon10b	TGTAAAACGACGGCCAGTtttggctataaaagatggactaca
BMI_exon10c	TGTAAAACGACGGCCAGTgacataacaggaaacagtattgtatga
BMI_exon10d	TGTAAAACGACGGCCAGTaagagttgcttttacagttaacaatg
BMI_exon1a	TGTAAAACGACGGCCAGTggcctgactacaccgacact
BMI_exon1b	TGTAAAACGACGGCCAGTCAGCCCCGCAGAATAAAAC
BMI_exon2&3	TGTAAAACGACGGCCAGTttgtgattactagatgatctccattc
BMI_exon4&5	TGTAAAACGACGGCCAGTagacagcatcacaatcaagtaaaa
BMI_exon6&7	TGTAAAACGACGGCCAGTttgactttaccacttccatcctc
BMI_exon8&9	TGTAAAACGACGGCCAGTgaggttggtcacctccaattt
BMI1_up_a	TGTAAAACGACGGCCAGTCGATTTGAACATGAGGTATTTC
BMI1_upstr_b	TGTAAAACGACGGCCAGTGCTTCTTACACAGCGCCATA
FAS_alt_upstr_a	TGTAAAACGACGGCCAGTAGTCTACTGAAAGGTGGAACAGA
FAS_alt_upstr_b	TGTAAAACGACGGCCAGTCCTAAGAGCTATCTACCGTTCCA

FAS_upstr_a	TGTAAAACGACGGCCAGTGAAGAAGGAGAAAGGCAGGA
FAS_upstr_b	TGTAAAACGACGGCCAGTCCAACCCTTTGACATTAGCA
MCL1_exon1a	TGTAAAACGACGGCCAGTcgcccctttccccttt
MCL1_exon1b	TGTAAAACGACGGCCAGTccccgtccaccctcac
MCL1_exon2	TGTAAAACGACGGCCAGTgaatcttgcgccggtttag
MCL1_exon3b	TGTAAAACGACGGCCAGTgcactaagaatgccagtgacc
MCL1_upstr_a	TGTAAAACGACGGCCAGTCCCTGCCCACAAAACATT
MCL1_upstr_b	TGTAAAACGACGGCCAGTGAGATGGGAGAAGCAAGCAG
MDM2_alt_upstr	TGTAAAACGACGGCCAGTCGGATGGTGAGGAGCAG
MDM2_exon11a	TGTAAAACGACGGCCAGTagcattcctgtgactgagca
MDM2_exon11b	TGTAAAACGACGGCCAGTcaattcaaatgattgtgctaactt
MDM2_exon2	TGTAAAACGACGGCCAGTtgatgtattttccacagatgtttc
MDM2_exon4	TGTAAAACGACGGCCAGTcctggttgtttacccctattca
MDM2_exon6&7	TGTAAAACGACGGCCAGTaaattgcataagggtttgtgtt
MDM2_exon8	TGTAAAACGACGGCCAGTggtactcaaaacagctcaattttc
MDM2_upstr_a	TGTAAAACGACGGCCAGTGAAGCGGGAGAAGGGAAG
MDM2_upstr_b	TGTAAAACGACGGCCAGTCGGCCTACCCAAAGTGA
miR15_upstr_A	TGTAAAACGACGGCCAGTTATTTCCGGTCCATTTACCA
miR15_upstr_B	TGTAAAACGACGGCCAGTtttctcatctcagggtaccaaa
miR155_loop	TGTAAAACGACGGCCAGTTGTACTGTGTCAGAATGCAAGC
miR155_upstr	TGTAAAACGACGGCCAGTCATACGTTTCTTTTGAACTTAGTCAC
miR17_upstr_A	TGTAAAACGACGGCCAGTCCCGGTCTTCTGTTCCTAAA
miR17_upstr_B	TGTAAAACGACGGCCAGTCAAATTTTTCTTGCTATTAAAGTTGA
PMAIP1_exon1&2	TGTAAAACGACGGCCAGTCCTACGTCACCAGGGAAGTT
PMAIP1_exon3a	TGTAAAACGACGGCCAGTTGGGCGTATTAGGTTTTGCT
PMAIP1_exon3b	TGTAAAACGACGGCCAGTGCTGGGGAGAAACAGTTCAG
PMAIP1_exon3c	TGTAAAACGACGGCCAGTAGTGTGCATCCTACAAAAAGTGA
PMAIP1_upstr_a	TGTAAAACGACGGCCAGTCAAGCAACTGGGACGACAG
PMAIP1_upstr_b	TGTAAAACGACGGCCAGTTCAAACACGATGTTCTTTCTGG
PUMA_exon1	TGTAAAACGACGGCCAGTggcagtcaagtttgagaagtctg
PUMA_exon2	TGTAAAACGACGGCCAGTcatgcacacacacctccact
PUMA_exon3	TGTAAAACGACGGCCAGTgcgatgggatcactagaaatg
PUMA_exon4a	TGTAAAACGACGGCCAGTcccactcccatcaccatact
PUMA_exon4b	TGTAAAACGACGGCCAGTctctcggtgctccttcactc
PUMA_upstr_b	TGTAAAACGACGGCCAGTGGCGGATCCCGAGGT
RFWD2_alt_upstr_a	TGTAAAACGACGGCCAGTTGCCATTAGACTTTACATTCCTG
RFWD2_alt_upstr_b	TGTAAAACGACGGCCAGTCGTAGACCTGAATTTGAATTTTGA
RFWD2_upstr_a	TGTAAAACGACGGCCAGTTTTCCGACTGGTGTCACCTT
RFWD2_upstr_b	TGTAAAACGACGGCCAGTAAAAAGCCTACCAGAAATTGTATATT
SKP2_alt_upstr	TGTAAAACGACGGCCAGTCGAGCAGCTCTGCAGTTAAT
SKP2_upstr_a	TGTAAAACGACGGCCAGTTTTCACTACAGAGCGAATCATTTT
SKP2_upstr_b	TGTAAAACGACGGCCAGTCGTCCACAGACCCAATTCTT

Amplicon Name	Reverse primer
BAX_exon1	CAGGAAACAGCTATGACcccctcagtgcttggagat
BAX_exon2&3	CAGGAAACAGCTATGACttaggggaggaggagaatgc
BAX_exon4	CAGGAAACAGCTATGACatccccaggtcctcacagat
BAX_exon5	CAGGAAACAGCTATGACccaatacggagtaatgccatc
BAX_exon6	CAGGAAACAGCTATGACgaacgggcattaaagagctg
BAX_exon7	CAGGAAACAGCTATGACgacagggcctgctctcct
BAX_upstr_b	CAGGAAACAGCTATGACGGACCCGTCCATCACC
BCL10_alt_upstr	CAGGAAACAGCTATGACCAAACGCACTCATACACACA
BCL10_upstr_b	CAGGAAACAGCTATGACCCGATTTACTGAACCATCCTT
BCL2_upstr_a	CAGGAAACAGCTATGACGGCTGCGCACCCTTTC
BCL2_upstr_b	CAGGAAACAGCTATGACGAGGATTTCCAGATCGATTCC
BCL6_exon1	CAGGAAACAGCTATGACttggcaagagcggaaaaa
BCL6_exon10a	CAGGAAACAGCTATGACtcgagcctttaacgcagttt
BCL6_exon10b	CAGGAAACAGCTATGACcctccaagagctttttccac
BCL6_exon2	CAGGAAACAGCTATGACcagtggcaaagttcacagaga
BCL6_exon3	CAGGAAACAGCTATGACcgtcatcccagatgcagtaa
BCL6_exon4	CAGGAAACAGCTATGACaaggtttctgatcggggact
BCL6_exon5a	CAGGAAACAGCTATGACaaatgcagggcaatctcatc
BCL6_exon5b	CAGGAAACAGCTATGACcaggcaagaaaagaagaaacg
BCL6_exon6	CAGGAAACAGCTATGACtgaggactgctccaatcaga
BCL6_exon7	CAGGAAACAGCTATGACaggatcctctccctgtcctg
BCL6_exon8	CAGGAAACAGCTATGACACTTTCTCCTGCCCGCTCT
BCL6_exon9	CAGGAAACAGCTATGACGTGGTTGCCCCACTGTGT
BCL6_upstr_a	CAGGAAACAGCTATGACTTTTGCCTTTTGCAAGTTTGT
BCL6_upstr_b	CAGGAAACAGCTATGACTTCGAGGTCCTCATAAGCAGA
BECN1_exon1&2	CAGGAAACAGCTATGACgccaccataagaattatatcacca
BECN1_exon10	CAGGAAACAGCTATGACgccaatatcaaagcaaacca
BECN1_exon11	CAGGAAACAGCTATGACtgctgcttcaattctgttcaa
BECN1_exon12a	CAGGAAACAGCTATGACtttttcagagaagaaagggaaa
BECN1_exon12b	CAGGAAACAGCTATGACcagaatttccacatacctgcat
BECN1_exon3	CAGGAAACAGCTATGACgccaagtagcctgcagaaac
BECN1_exon5&6&7	CAGGAAACAGCTATGACggaagccatttcctctcctg
BECN1_exon8	CAGGAAACAGCTATGACacccaggcttagtggtcaac
BECN1_exon9	CAGGAAACAGCTATGACtggacagcagtaccaggttg
BECN1_upstr_a	CAGGAAACAGCTATGACGCAAGGCTCCATCTCAGAAC
BECN1_upstr_b	CAGGAAACAGCTATGACCCCGATGCTCTTCACCTC
BIM_upstr_a	CAGGAAACAGCTATGACAAGTGCGTCACAATAGAGTTTG
BIM_upstr_b	CAGGAAACAGCTATGACACCTTCTCGGTCACACTCAG
BMI_exon10a	CAGGAAACAGCTATGACgaaaccatttcccatctttcc
BMI_exon10b	CAGGAAACAGCTATGACgggtatctgcaaaggtcgaa
BMI_exon10c	CAGGAAACAGCTATGACttttaaattcaacatgcttgctc
BMI_exon10d	CAGGAAACAGCTATGACgccacatcactataagtcagcaa
BMI_exon1a	CAGGAAACAGCTATGACCCACGCTCGTCTCATGC
BMI_exon1b	CAGGAAACAGCTATGACagcggggaactaaaacgtg
BMI_exon2&3	CAGGAAACAGCTATGACgaaataaagagggttgccttca
BMI_exon4&5	CAGGAAACAGCTATGACgaggatggaagtggtaaagtcaa
BMI_exon6&7	CAGGAAACAGCTATGACgggcaacacaatccctgata
BMI_exon8&9	CAGGAAACAGCTATGACtggaagtggaccattctacaaa
BMI1_up_a	CAGGAAACAGCTATGACTCGCATCGTTTCCTCCGTGT
BMI1_upstr_b	CAGGAAACAGCTATGACCAAACGCACTCATACACACA
FAS_alt_upstr_a	CAGGAAACAGCTATGACTTTCCTGGCCAATTTTCTTT
FAS_alt_upstr_b	CAGGAAACAGCTATGACGTCAGGAGGATGGGAAGGA

FAS_upstr_a	CAGGAAACAGCTATGACTATGTGCTTTGTGGGGAGAC
FAS_upstr_b	CAGGAAACAGCTATGACGGCTTGTCTCTGTTCCACCT
MCL1_exon1a	CAGGAAACAGCTATGACggcgcgaagaaaagcag
MCL1_exon1b	CAGGAAACAGCTATGACgactcgtttcggtttccaac
MCL1_exon2	CAGGAAACAGCTATGACcctttagatatccccacctctc
MCL1_exon3b	CAGGAAACAGCTATGACtgagtattgcccaatcagagc
MCL1_upstr_a	CAGGAAACAGCTATGACGCTCCACGTGCTACCCTAAA
MCL1_upstr_b	CAGGAAACAGCTATGACGAAGCGGAAGTGAGAAGTGG
MDM2_alt_upstr	CAGGAAACAGCTATGACGCACCATCAGTAGGTACAGACA
MDM2_exon11a	CAGGAAACAGCTATGACcaggttgtctaaattcctagggtta
MDM2_exon11b	CAGGAAACAGCTATGACgcgacagagagactcatctcaa
MDM2_exon2	CAGGAAACAGCTATGACgagccatgctacaattgaggt
MDM2_exon4	CAGGAAACAGCTATGACggtagaggtgaactgaaatgttagc
MDM2_exon6&7	CAGGAAACAGCTATGACgggaacaatttaattaagtctaaaagc
MDM2_exon8	CAGGAAACAGCTATGACtccttattaggactgccagga
MDM2_upstr_a	CAGGAAACAGCTATGACAACGAAGATGCTGGTTACCG
MDM2_upstr_b	CAGGAAACAGCTATGACGCTTCTTGCTCCATCTTTCC
miR15_upstr_A	CAGGAAACAGCTATGACTCTTAGAGTTGGATATCATTTGATACAGT
miR15_upstr_B	CAGGAAACAGCTATGACtgcataattatttttaaggagattttaag
miR155_loop	CAGGAAACAGCTATGACTTCTGTTAATGATAAAAACAAACATGG
miR155_upstr	CAGGAAACAGCTATGACAAACCGCTCTTCGGAGTTTT
miR17_upstr_A	CAGGAAACAGCTATGACGCAAATCCGTACTTAATGAGTTAGTTT
miR17_upstr_B	CAGGAAACAGCTATGACTGACTGGTCACAATCTTCAGTT
PMAIP1_exon1&2	CAGGAAACAGCTATGACCTGGAAGCGCTGGGAGTC
PMAIP1_exon3a	CAGGAAACAGCTATGACTTTCAGTGTTCTCAGAATGAGTTTTT
PMAIP1_exon3b	CAGGAAACAGCTATGACTCACCAATGCATATATGGAACTTT
PMAIP1_exon3c	CAGGAAACAGCTATGACCAGAGGATGTCTGCTGATGG
PMAIP1_upstr_a	CAGGAAACAGCTATGACACCTTAATCACCGCAGAGGA
PMAIP1_upstr_b	CAGGAAACAGCTATGACGATGCTGGGATCGGGTGT
PUMA_exon1	CAGGAAACAGCTATGACcacctgtgacagcttcttgct
PUMA_exon2	CAGGAAACAGCTATGACcagttcctccgagtctccag
PUMA_exon3	CAGGAAACAGCTATGACagctgccgcacatctgg
PUMA_exon4a	CAGGAAACAGCTATGACctggagcaaccggcaaac
PUMA_exon4b	CAGGAAACAGCTATGACctctcccacccactcacaag
PUMA_upstr_b	CAGGAAACAGCTATGACACCCCATGCCAAATTTCAT
RFWD2_alt_upstr_a	CAGGAAACAGCTATGACTTTCAACAACAAAGTGAAATAGGT
RFWD2_alt_upstr_b	CAGGAAACAGCTATGACGTGGCCACATTTTGTCATGT
RFWD2_upstr_a	CAGGAAACAGCTATGACCAATAATGGGAAAACAGATTTTG
RFWD2_upstr_b	CAGGAAACAGCTATGACAGAGGGCAGGCCGTCTTAAT
SKP2_alt_upstr	CAGGAAACAGCTATGACGCCTGACAGCAGTTCAGAAG
SKP2_upstr_a	CAGGAAACAGCTATGACAGGTGAGAGGCTGCGAACT
SKP2_upstr_b	CAGGAAACAGCTATGACGCAGCCAGACCCGCTAAG
