# Supplementary material for: Genetic Variation in Cell Death Genes and Risk of Non-Hodgkin Lymphoma
Source: PLoS One. 2012 Feb 7;7(2):e31560. doi: 10.1371/journal.pone.0031560 (PMC3274532; doi:10.1371/journal.pone.0031560)
Supplement: Table S4 — Variants available for testing after data cleaning. (PDF) [file pone.0031560.s004.pdf]

Table S4 - Variants available for testing after data cleaning

BAX	
rs11667229	
rs11667351	
rs4645878	
rs1805419	
rs4645900	
rs704243	
rs905238	
BCL2	
rs4987873	
rs4987853	
rs4987852	
rs1564483	
rs4987845	
rs1982673	
rs17677569	
rs10503078	
rs4456611	
rs1026825	
rs7230970	
rs4941185	
rs7240326	
rs7226979	
rs7236090	
rs17759659	
rs2850761	
rs1462129	
rs1801018	
rs2279115	
rs1944423	
BCL6	
rs1474326	
rs2229362	
rs41268621	
rs1056932	
rs2272148	
BCL6-x8(2139)-C*T	
rs3733017	
BCL6-x10(3083)-A*T	
rs1523474	
rs3774298	
rs4686467	
BCL10	
rs962409	

rs12134420<br>rs12563515<br>rs4949927<br>rs4949928<br>rs2735594<br>rs11161582	
BCL2L11<br>rs17041868<br>rs10204044<br>rs17484848<br>rs3761704<br>rs1877331<br>rs17041883<br>rs1470053	
BBC3<br>rs884171<br>rs45474992<br>rs2032809	
BECN1<br>rs10512488	
BMI1<br>rs985000<br>BMI1-UPSTR(-809)-C*T	
FAS<br>rs983751<br>rs978522<br>rs1926197<br>rs3758483<br>rs2234767<br>rs10509561<br>rs1571013<br>rs2147420<br>rs1159120<br>rs9658750<br>rs9658761<br>rs982764<br>rs2234978<br>rs1051070	
MCL1<br>rs6655975<br>rs11204666<br>rs878471<br>rs35392872<br>rs34355066<br>MCL1-x3(1290-1297)-DEL(aacctagc)<br>rs34645101<br>rs35661734	 <br><br><br><br><br>*

MDM2	
rs937283	
rs2870820	
rs3730536	
rs1625525	
rs1695144	
rs1695147	
rs769412	
PMAIP1	
rs9957673	
rs11663656	
PMAIP1-x2(422)-G*A	
rs7240884	
rs1041978	
rs3826598	*
rs4286195	
RFWD2	
rs6676805	
rs12041226	
rs12142010	
rs2481641	
rs617078	
rs11587785	
rs12144612	
rs12738115	
RFWD2-UPSTR(-184)-C*G	
RFWD2-IVS2(-297)-A*G	
SKP2	
rs10071838	
rs33671	
rs12655652	
rs4440390	
rs33678	
rs2362973	
rs7715070	
rs2287935	
rs3804439	
rs17279275	
rs7731023	
MIR15A	
rs2476391	
miR15a-UPSTR(-1296)-A*G	
rs9535416	
MIR1792	
rs17642969	
MIR155	
rs972389	

rs928883	
rs2829803	

* denotes SNPs that passed quality control measures but were not analyzed because there were insufficient samples possessing the minor allele.
