# Supplementary material for: Genetic Variation in Cell Death Genes and Risk of Non-Hodgkin Lymphoma
Source: PLoS One. 2012 Feb 7;7(2):e31560. doi: 10.1371/journal.pone.0031560 (PMC3274532; doi:10.1371/journal.pone.0031560)
Supplement: Table S7 — Interaction table displaying odds ratios for rs928883 and two AICDA SNPs, rs714629 and rs9971686, derived under an additive model. (PDF) [file pone.0031560.s007.pdf]

Table S7 - Interaction table displaying odds ratios for rs928883 and two AICDA SNPs, rs714629 and rs9971686, derived under an additive model

rs928883	rs714629			rs9971686		
	CC	CG	GG	AA	AG	GG
GG	1.00	0.42	0.18	1.00	0.26	0.07
GA	2.80	0.95	0.32	2.80	0.82	0.24
AA	7.84	2.13	0.58	7.84	2.56	0.83
